# Risk factors for severe opioid-induced respiratory depression in hospitalized adults: A case–control study

**DOI:** 10.1080/24740527.2020.1714431

**Published:** 2020-05-21

**Authors:** Madalina Boitor, Ariane Ballard, Jessica Emed, Sylvie Le May, Céline Gélinas

**Affiliations:** a Faculty of Dentistry, McGill University, Montreal, Quebec, Canada; b Faculty of Nursing, University of Montreal, Montreal, Quebec, Canada; c CIUSSS West-Central-Montreal, Jewish General Hospital, Montreal, Quebec, Canada; d Research Centre, CHU Sainte-Justine, Montreal, Quebec, Canada; e Ingram School of Nursing, McGill University, Montreal, Quebec, Canada; f Centre for Nursing Research and Lady Davis Institute, Jewish General Hospital, Montreal, Quebec, Canada

**Keywords:** opioids, respiratory depression, adult, hospitalized, case–control

## Abstract

**Background**: Opioids are commonly prescribed to hospitalized adults to promote adequate pain relief, yet they can cause potentially fatal respiratory depression.

**Aim**: The aim of this study was to examine the risk factors for the development of severe opioid-induced respiratory depression (OIRD) in hospitalized adults to ensure adequate monitoring of high-risk patients.

**Methods**: A retrospective case–control study was conducted using data from the medical records of a university-affiliated hospital in Canada. Cases were eligible if they were adults (≥18 years old) and received opioid analgesia within 24 h of naloxone administration for respiratory depression. Controls had the same eligibility criteria, except for respiratory depression and naloxone administration. The case–control ratio was 1:1, and they were matched based on sex, type of unit, opioid molecule and the presence/absence of medication errors.

**Results**: A total of 133 cases and 133 controls were included. Following cumulative risk factor analysis, renal failure (odds ratio [OR] = 2.176, 95% confidence interval [CI], 1.021–4.640, *P* = 0.044), the first 24 h of opioid administration (OR = 1.899, 95% CI, 1.090–3.309, *P* = 0.024), concomitant central nervous system (CNS) depressants (OR = 1.785, 95% CI, 1.023–3.113, *P* = 0.041), and increasing age (OR = 1.019, 95% CI, 1.002–1.035, *P* = 0.028) were positively associated with severe OIRD.

**Conclusions**: Some adult hospitalized patients were at higher risk of experiencing severe OIRD, such as those with renal failure, those in their first 24 h of opioid administration, those receiving CNS depressants in addition to opioids, and those with an advanced age. These results will assist with the screening of patients at higher risk for severe OIRD, which is key to implementing appropriate monitoring and enhancing the safety of opioid use in hospital settings.

## RÉSUMÉ

**Contexte**: Les opioïdes sont couramment prescrits aux adultes hospitalisés pour favoriser un soulagement adéquat de la douleur, mais ils peuvent provoquer une dépression respiratoire potentiellement mortelle.

**Objectif**: Examiner les facteurs de risque du développement d'une dépression respiratoire sévère induite par un opioïde chez les adultes hospitalisés afin d'assurer un suivi adéquat des patients à haut risque.

**Méthodes**: Une étude rétrospective cas - témoins a été menée en utilisant les données des dossiers médicaux d'un hôpital universitaire au Canada. Les cas étaient admissibles s'ils étaient adultes (≥ 18 ans) et avaient reçu une analgésie opioïde dans les 24 heures suivant l'administration de naloxone pour une dépression respiratoire. Les critères d'admissibilité étaient les mêmes pour les témoins, à l’exception de la dépression respiratoire et de l'administration de naloxone. Le rapport cas/témoins était de 1:1 et ceux-ci étaient appariés en fonction du sexe, du type d'unité, de la molécule opioïde et de la présence ou absence d'erreurs de médication.

**Résultats**: Un total de 133 cas et 133 témoins ont été inclus. Après analyse des facteurs de risque cumulés, l'insuffisance rénale (RC = 2,176, IC à 95 % : 1,021 - 4,640, *p* = 0,044), les premières 24 heures d'administration d'opioïdes (RC = 1,899, IC à 95 % : 1,090 -3, 309, *p* = .024), les dépresseurs du SNC concomitants (RC = 1,785, IC à 95 % : 1,023-3,113, *p* = 0,041) et l'augmentation de l’âge par âge avancé (RC = 1,019, IC à 95 % : 1,002 - 1,035, *p* = 0,028) ont été positivement associés à des dépressions respiratoires sévères induites par un opioïde.

**Conclusions**: Certains patients adultes hospitalisés présentaient un risque plus élevé de souffrir d'une dépression respiratoire sévère induite par un opioïde, notamment les personnes souffrant d'insuffisance rénale, celles à qui un opoïde a été administré dans les dernières 24 heures, celles qui ont reçu ds dépresseurs du SNC en plus d'un opioïde et celles qui étaient plus âgées. Ces résultats permettront d'identifier les patients à haut risque de souffrir d'une dépression respiratoire sévère induite par un opioïde, ce qui est essentiel pour mettre en place un suivi approprié et améliorer la sécurité de l'utilisation des opioïdes en milieu hospitalier.

## Introduction

Opioids are commonly prescribed to hospitalized patients to relieve pain and prevent adverse consequences of unrelieved pain. Yet, the administration of opioids itself is known to be associated with adverse events such as sedation, respiratory depression, nausea, vomiting, and urticaria.^1–3^ Of these, respiratory depression (<8–10 breaths per minute) is one of the most serious adverse reactions post opioid administration.^4^ This adverse reaction is due to the interaction of opioids with the central nervous system (CNS) causing decreased respiratory drive, sedation, and upper airway obstruction.^5,6^ The incidence of opioid-induced respiratory depression (OIRD) in patients treated with opioids for acute postoperative pain is estimated at 0.5%,^7^ but it can occur in all patients receiving opioids. OIRD remains a significant cause of brain damage and death but, fortunately, most cases are preventable with better screening and monitoring.^8^

Even at comparable equianalgesic doses of opioids, not all patients experience respiratory depression. Some patients may be at higher risk for OIRD due to their intrinsic characteristics (e.g., advanced age [>60 years old], female sex, opioid naïvete, presence of multiple comorbidities)^9–11^ or iatrogenic factors such as undergoing surgery and concomitant administration of CNS depressants.^11,12^ If not detected and treated on time, OIRD can be fatal. Therefore, it is imperative to identify the specific factors that put patients at high risk of OIRD to ensure adequate screening and monitoring for these patients and prevent the development of OIRD.

Several studies have been conducted to identify potential risk factors for OIRD in the postoperative context and were summarized in recent review including 13 studies targeting patients post orthopedic, general, thoracic, transplant, urologic, gynecologic, and neurosurgeries.^12^ Most studies were conducted in the postanesthesia care unit, followed by the ward and the intensive care unit, and OIRD occurred mainly within the first 24 h postsurgery. The most important risk factors were age, female biological sex, American Society of Anesthesiologists classification III/IV, obstructive sleep apnea, chronic obstructive pulmonary disease, cardiac and neurological disease, concomitant administration of sedatives, use of patient-controlled anesthesia pump, multiple routes of administration, and multiple prescribers. Another study by Pawasauskas et al. with hospitalized adult patients on general medical units (65 cases and 65 controls) showed positive associations between naloxone administration and concurrent use of CNS depressant medication; renal, cardiac, and respiratory disease; and smoking status.^13^ Of note, patient records were excluded if naloxone was administered within 24 h of admission or was administered in the emergency department or postanesthesia care unit. Despite these informative results, none of the previous studies explored baseline risk factors for OIRD in all adult hospitalized patients to allow for risk stratification of patients receiving opioids.

This study aimed to identify the risk factors for the development of severe OIRD in hospitalized adults receiving prescribed opioids. Based on previous research, we hypothesized that all variables considered in this study had the potential to act as risk factors for the development of in-hospital OIRD. A secondary objective was to compare the 30-day mortality between cases and controls. We hypothesized that mortality would be higher in cases than in controls.

## Materials and methods

### Study design

A case–control design using retrospective data collection was used for this study. This article adheres to the Strengthening the Reporting of Observational Studies in Epidemiology guidelines for case–control studies.^14^

### Study setting

This study was conducted at a university-affiliated hospital setting in Montreal, Quebec, Canada. The research ethics committee and the director of professional services of the study site approved the conduct of this study (No. CODIM-MBM-CR17-38) and waived the need for informed consent as per article 19.2 of the Loi sur les services de santé et services sociaux du Québec. There was no direct contact with patients. Data extraction started in September 2017 and ended in April 2018.

### Sample

Potential cases and controls were screened from medical records dated from April 2009 to February 2018. Cases were screened from a list with patients who received naloxone during their hospitalization. Cases were considered eligible for inclusion if they were adults (18 years of age and older) and received opioid analgesia within 24 h of naloxone administration for respiratory depression. Those who received opioids prior to hospital admission (e.g., at home) were excluded given the lack of documentation and certitude of the type, dose, and timing of opioid intake. Additionally, those who received naloxone for purposes other than reversal of respiratory depression (e.g., pruritus) were excluded. One control was selected for each case and matched based on sex, type of unit (acute vs. critical care), type of opioid molecule, and presence/absence of medication error. Controls had the same inclusion criteria as cases, except for the lack of respiratory depression and naloxone administration. Controls were selected from a list with patients who received opioids during their hospital stay following the matching criteria. The list was generated by the hospital’s medical records personnel.

The G*Power 3 program^15^ was used to calculate the minimum sample size required to be able to detect a small effect size using an odds ratio (OR) of 1.5,^16^ a power of 80%, and level of significance of 5%. A minimum sample size of 254 patients (total) was needed to meet the study’s objective.

### Data extraction

Demographic (i.e., age, sex) and clinical data were extracted from medical records for eligible cases and controls by two doctoral trainees with a clinical background. Clinical data included information about the reason for admission, the unit where patients were admitted, habits, preexisting comorbidities, and in-hospital variables including the administration of opioids and CNS depressants (Supplemental Digital Content File 1). The potential risk factors examined in this study were based on the American Society for Pain Management Nursing guidelines for monitoring opioid-induced respiratory sedation and depression.^11^

### Statistical analyses

SPSS (version 22.0; IBM Corporation, Armonk, NY) software was used for data analysis. Data are summarized using frequencies and percentages for nominal variables and median and interquartile ranges (IQRs) for continuous nonnormally distributed variables. Variables with extensive missing data (>75%) are summarized but not are included in comparisons between cases and controls or in logistic regression.

Pearson’s chi-square test was used to compare the distribution of nominal variables between cases and controls, and the Mann-Whitney *U* test was used for continuous variables. Risk factors were determined based on comparisons of the incidence of each predefined risk factor in case patients with the corresponding incidence in the control patients using logistic regression. Estimates of odds ratios and accompanying 95% confidence intervals (CIs) were calculated for every risk factor. Then, cumulative risk factor analysis was performed in which all variables identified as risk factors were included except for those with a high incidence of missing data. A new variable was created based on the sum of all risk factors present (*P* < 0.05) for each patient. Bar charts are used to display the distribution of cases and controls by the number of risk factors and age groups.

## Results

**Figure 1.**
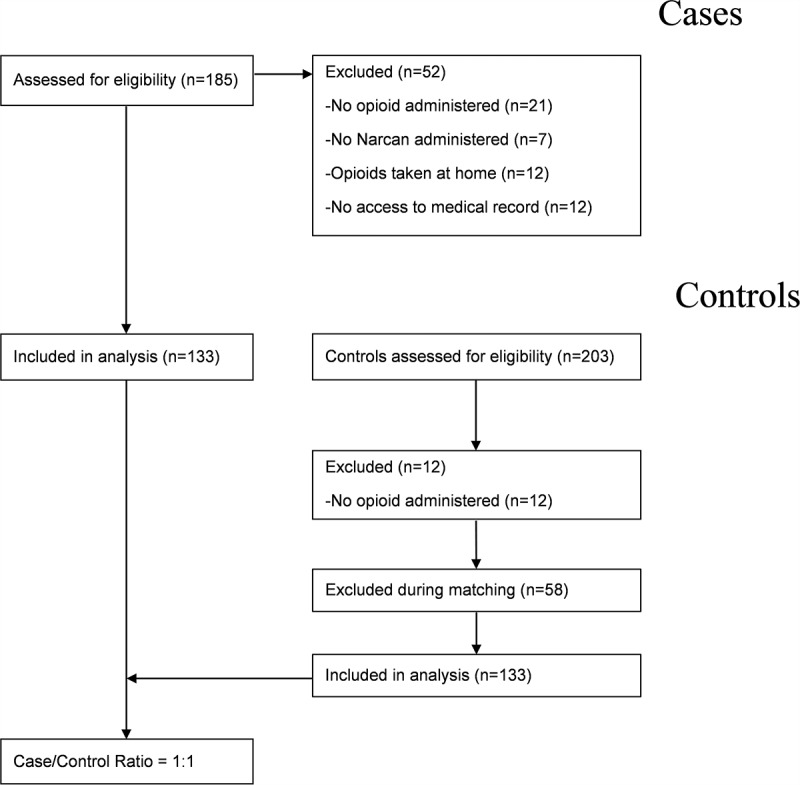
Participant flow diagram

A total of 185 medical records were screened for inclusion as cases and 203 for inclusion as controls (Figure 1). During the screening time frame, a total of 133 adult patients had experienced respiratory depression with subsequent administration of naloxone as bolus (*n* = 126) or drip (*n* = 7) following in-hospital opioid administration. Of these, 21 (16%) had documentation of a respiratory rate ≤8 associated with moderate or important drowsiness, and 15 had a desaturation ≤92% associated with a respiratory rate ≤8 and moderate or important drowsiness. All cases had a physician diagnosis of OIRD. Of the 133 cases, 82 were female (62%; Table 1). Overall, patients experiencing severe respiratory depression had a median age of 73 compared to controls, who had a median age of 65 (Mann-Whitney = 6689.5, *P* = 0.001).

**Table 1.** Sample demographic and clinical characteristics

Variable	Cases (*n* = 133)	Controls (*n* = 133)	Total (*n* = 266)	Statistical comparison between groups
Age (median, range)	73 (19–96)	65 (20–97)	—	Mann-Whitney = 6689.50, *P* = 0.001
Reason for admission (*n*)				χ^2^ = 10.24, *P* = 0.176
Orthopedic	29	28	57	
Cardiac	24	29	53	
Cancer	27	17	44	
Gastrointestinal/genitourinary	20	23	43	
Neurologic	12	11	23	
Pulmonary	10	8	18	
Elective surgery	6	2	8	
Other	5	15	20	
Medical context (*n*)				χ^2^ = 0.031, *P* = 0.859
Acute care	114	115	229	
Critical care	19	18	37	
Medical error (*n*)				χ^2^ = 228.17, *P* < 0.001
Incorrect dose	11	11	22	
Incorrect route	3	3	6	
Incorrect drug	1	4	5	
Without prescription	2	1	3	
Incorrect patient	1	—	1	
Incorrect drug and route	1	—	1	
Discontinued	1	1	2	
Equianalgesia in milligrams of morphine per os over 24 h (median, range)	50 (1.25–2550)	50 (5–480)	—	Mann-Whitney = 8637.50, *P* = 0.741

**Figure 2.**
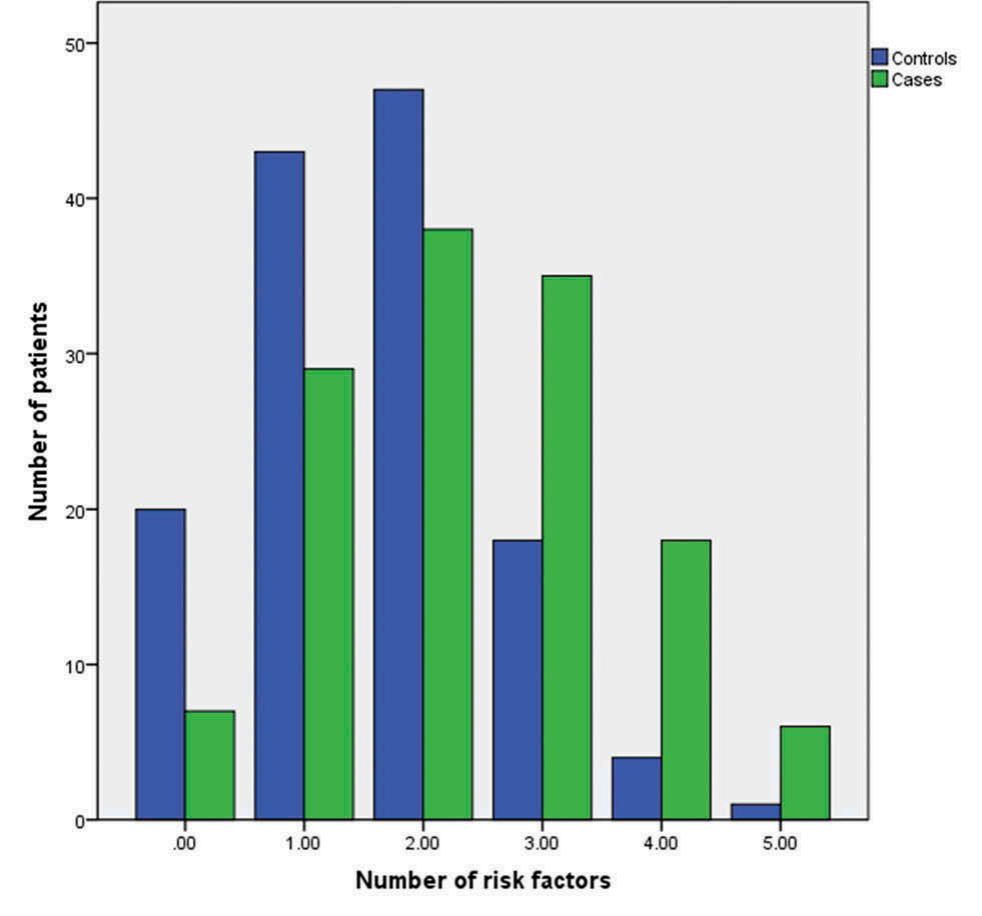
Distribution of patients according to the occurrence of risk factors (i.e., respiratory, cardiac, renal, hepatic, first 24 h of opioids, and CNS depressants)

The variables retrognathia and neck circumference had substantial missing data and were excluded from analyses. The prevalence of predefined risk factors in cases and controls and their individual ORs are displayed in the Supplemental Digital Content File 2. Based on a level of significance <0.05, seven risk factors were identified for the development of severe OIRD, namely, age (OR = 1.025, 95% CI, 1.01–1.04), respiratory (OR = 1.719; 95% CI, 1.03–2.87) and cardiac disease (OR = 1.911, 95% CI, 1.15–3.16), renal (OR = 3.046, 95% CI, 1.52–6.10) and liver (OR = 2.691, 95% CI, 1.01–7.17) failure, first 24 h of opioid administration (OR = 1.774, 95% CI, 1.06–2.96), and concomitant CNS depressants (OR = 1.842, 95% CI, 1.10–3.09). Using the sum of these dichotomous risk factors and excluding age, cases (median = 2, IQR = 2; mean = 2.35, SD = 1.24) had significantly more risk factors than controls (median = 2, IQR = 1; mean = 1.59, SD = 1.04; Mann-Whitney = 5828.50, *P* < 0.001), with differences more pronounced for patients with three, four, and five risk factors (Figure 2).

**Figure 3.**
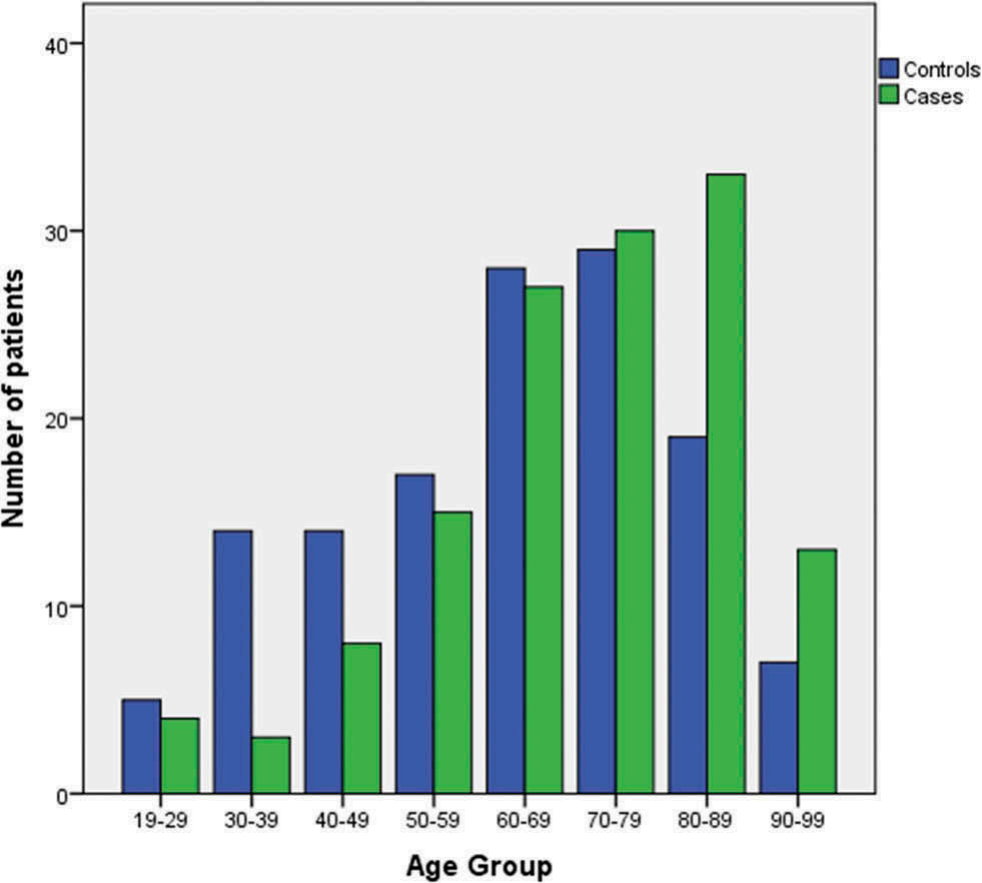
Distribution of case and control patients according to age groups

For cumulative risk factor analysis, a logistic regression was performed to ascertain the effects of age, respiratory and cardiac disease, renal and liver failure, first 24 h of opioid administration, and concomitant CNS depressants on the likelihood of developing severe OIRD and receiving naloxone in hospitalized patients. The logistic regression model was statistically significant, χ^2^(7) = 34.00, *P* < 0.001, explained 16.0% (Nagelkerke’s *R*^2^) of the variance in OIRD, and correctly classified 65.8% of cases. Advanced age was associated with an increased likelihood of experiencing severe OIRD (OR = 1.019, 95% CI, 1.002–1.035, *P* = 0.028; Figure 3). Patients diagnosed with renal failure were 2.18 times as likely to experience OIRD than those not diagnosed with renal failure (OR = 2.176, 95% CI, 1.021–4.640, *P* = 0.044). Patients in their first 24 h of opioid administration were 1.90 times as likely to experience severe respiratory depression than those who had previously received opioids (OR = 1.899, 95% CI, 1.090–3.309, *P* = 0.024). Those who received CNS depressants in addition to opioids were 1.79 times as likely to experience severe respiratory depression than those who did not (OR = 1.785, 95% CI, 1.023–3.113, *P* = 0.041).

Several potential risk factors such as past naloxone administration (OR = 5.156), substance use disorder (OR = 2.047), sleep apnea (OR = 1.792), alcohol use disorder (OR = 1.260), and increased opioid dosage (>50%; OR = 2.019) were rare in our sample but appear to be strongly associated with severe OIRD.

A total of 34 (13%) patients died in the 30 days following the data extraction time frame. Thirty-day mortality was significantly higher in cases (*n* = 28) than in controls (*n* = 6), χ^2^ = 16.32, *P* < 0.001.

## Discussion

This study identified risk factors for the development of severe OIRD in hospitalized adults. The strongest predictors of severe OIRD were renal and liver failure, concomitant administration of CNS depressants such as benzodiazepines, first 24 h of opioid administration, cardiac and respiratory disease, and advanced age. Following cumulative risk factor analysis, only renal failure, first 24 h of opioid administration, concomitant CNS depressants, and age were significantly associated with severe OIRD.

Renal and liver failure appeared to be strong predictors of severe OIRD, with ORs above 2.5. Such findings are expected, because patients with these conditions have impaired metabolism and excretion of opioids. An accumulation of opioids and their toxic metabolites in patients with renal failure may lead to severe oversedation and suppression of respiratory drive.^17,18^ Patients with liver failure have a decreased metabolism and increased risk for accumulating opioids in the body, especially with frequent administrations.^19^ The first 24 h of opioid administration was also a strong independent risk factor for severe OIRD. Most patients experience sedation at the beginning of opioid therapy and are at risk for developing clinically significant respiratory depression after receiving their first dose of opioids.^20^

The concomitant administration of CNS depressants and opioids may have an interactive or synergistic effect, thereby increasing the risk for respiratory depression, as seen in this study. Benzodiazepines were the most frequently prescribed CNS depressants (24% of patients) and the only ones that were statistically significant risk factors for severe OIRD. Benzodiazepines are the most widely prescribed psychoactive drugs, are used by twice as many females than males, and are commonly prescribed for people in their 60s and 70s.^21^ They have precedence over other psychoactive drugs given their safety profile, but they are known to generate adverse events such as drowsiness and dizziness.^21^ The respiratory depressant effect of benzodiazepines is generally mild, yet their concurrent use with opioids can increase and prolong the respiratory depressant effects of opioids.^22^ In a previous study on naloxone use in hospitalized patients, patients receiving benzodiazepines and opioids were significantly more likely to require respiratory depression reversal using naloxone than those who had not received any benzodiazepines.^23^ The combination of opioids with benzodiazepines is advantageous for the anxiolytic and skeletal muscle relaxant effect but requires closer monitoring to avoid respiratory depression and subsequent adverse events.

Similar results were reported in a study of 130 adult hospitalized patients admitted to any unit, where renal, cardiac and respiratory disease, smoking status, and concurrent use of CNS depressants were significantly associated with naloxone administration.^13^ In our study, past and/or current smoking was not associated with severe OIRD and was documented in only 24% of the charts compared to the 47% prevalence in the study by Pawasauskas et al.^13^ Several studies investigated the risk factors for postoperative OIRD and identified older age (>65), female sex, presence of comorbidities (e.g., obstructive sleep apnea, renal disease, chronic obstructive pulmonary disease, cardiac disease, diabetes mellitus, neurologic disease, obesity, opioid dependence), use of patient-controlled analgesia, multiple prescribers of opioids, and use of two or more opioids as associated with OIRD.^12,24–26^ Many of these were significant risk factors in our study, although only 30% of our sample included postoperative patients.

In addition to the aforementioned risk factors, past naloxone administration, substance use disorder, sleep apnea, and alcohol use disorder may be important predictors for OIRD but were not frequent in our study sample (*n* < 12) and thus lacked statistical significance. Similarly, increased opioid dosage (>50%) appears to be strongly associated with severe OIRD but was not statistically significant (*P* > 0.10) because only 26 patients had an increase in dosage. Diagnosed substance use disorder (OR = 10.20, 95% CI, 9.06–11.40) and higher daily opioid use (OR = 2.31, 95% CI, 1.90–2.81) have been shown to be strongly associated with OIRD in medical users of prescription opioids in a large study with 7234 cases and 28,932 controls,^27^ which underscores their relevance in predicting OIRD. Sleep apnea is an important risk factor for OIRD^12,28–30^ and should be considered in the estimation of the risk of OIRD for each patient.

Several potential protective factors were identified in this study but were nonsignificant given their rare occurrence in the study population. As expected, a lower American Society of Anesthesiologists score was associated with a decreased risk of severe OIRD. The concomitant administration of long- and short-acting medications appeared to be protective, possibly due to the longer-term opioid therapy for these patients and the tolerance developed over time, especially because only 6% of our study sample were in their first 24 h of opioid administration, and this combination of analgesics is common for chronic noncancer pain.^31^

The 30-day mortality rate for patients receiving naloxone (21%) is consistent with current reports of naloxone’s success in reversing respiratory depression (>75%),^32^ although preexisting medical conditions could have accounted for some of these deaths. Mortality was almost five times more frequent in cases than controls, which shows the possible fatality associated with OIRD and calls for strategies to prevent OIRD in hospitalized adults.

In most cases, OIRD occurs without obvious overdose of opioids but rather is secondary to drug-related factors (e.g., doses of administration) and/or patient-related characteristics (e.g., comorbidities) that enhance the CNS depressant effects of opioids or results in opioid accumulation or excessive duration of action.^29^ Identification of patients at risk of OIRD can allow for better risk stratification, timely response, and efficient resource allocation. Research efforts are currently devoted to the development of opioids without CNS depressant effects,^33^ but until they become routinely prescribed in hospital settings, adequate monitoring following opioid administration remains one of the best currently available strategies for preventing OIRD.^8,34^

### Limitations

The results of this study are based on retrospective data extraction from medical records at the study site, where documentation by exception is the norm. We assumed that the lack of documentation of existing risk factors (e.g., cardiac or renal disease) in any medical file corresponded to the absence of the respective risk factors. This assumption might have underestimated the prevalence of risk factors in both cases and controls, thereby minimizing the bias in the estimation of ORs. Furthermore, naloxone administration was used as surrogate for severe OIRD, and cases with less severe forms of OIRD that did not involve the administration of naloxone were omitted in this study. Therefore, it is possible that additional risk factors other than those identified in the present study may pose a risk for the development of opioid-induced adverse events (e.g., oversedation not requiring naloxone).

### Conclusion

This study helped outline risk factors for in-hospital severe OIRD and showed that mortality is more frequent in cases than controls. Comorbidities (i.e., renal failure), the first 24 h of opioid administration, concomitant use of benzodiazepines, and advanced age are risk factors independently associated with severe OIRD. Findings will assist with the screening process to identify patients at high risk of severe OIRD and in subsequent implementation of appropriate monitoring to enhance the safety of opioid use in hospital settings.

## Supplementary Material

Supplemental Material

Supplemental Material

## References

[1] de Boer HD, Detriche O, Forget P. Opioid-related side effects: postoperative ileus, urinary retention, nausea and vomiting, and shivering. A review of the literature. Best Pract Res-Clin. 2017;31(4):499–504. doi:10.1016/j.bpa.2017.07.002. 29739538

[2] Benyamin R, Trescot AM, Datta S, Buenaventura R, Adlaka R, Sehgal N, Glaser SE, Vallejo R. Opioid complications and side effects. Pain Physician. 2008;11:S105–120. 18443635

[3] Imam MZ, Kuo A, Ghassabian S, Smith MT. Progress in understanding mechanisms of opioid-induced gastrointestinal adverse effects and respiratory depression. Neuropharmacology. 2018;131:238–55. doi:10.1016/j.neuropharm.2017.12.032. 29273520

[4] Pasero C, Quinn TE, Portenoy R, McCaffery M, Rizos A. Opioid analgesics. In: Pasero C, McCaffery M, editors. Pain assessment and pharmacologic management. St. Louis (MO): Mosby/Elsevier; 2011. p. 277–622.

[5] Macintyre PE, Loadsman JA, Scott DA. Opioids, ventilation and acute pain management. Anaesth Intensive Care. 2011;39(4):545–58. doi:10.1177/0310057X1103900405. 21823370

[6] Dahan A, Sarton E, Teppema L, Olievier C, Nieuwenhuijs D, Matthes HWD, Kieffer BL. Anesthetic potency and influence of morphine and sevoflurane on respiration in mu-opioid receptor knockout mice. Anesthesiology. 2001;94(5):824–32. doi:10.1097/00000542-200105000-00021. 11388534

[7] Dahan A, Aarts L, Smith TW, Incidence R. Prevention of opioid-induced respiratory depression. Anesthesiology. 2010;112(1):226–38. doi:10.1097/ALN.0b013e3181c38c25. 20010421

[8] Lee LA, Caplan RA, Stephens LS, Posner KL, Terman GW, Voepel-Lewis T, Domino KB. Postoperative opioid-induced respiratory depression a closed claims analysis. Anesthesiology. 2015;122(3):659–65. doi:10.1097/ALN.0000000000000564. 25536092

[9] Cepeda MS, Farrar JT, Baumgarten M, Boston R, Carr DB, Strom BL. Side effects of opioids during short-term administration: effect of age, gender, and race. Clin Pharmacol Ther. 2003;74(2):102–12. doi:10.1016/S0009-9236(03)00152-8. 12891220

[10] Davies EC, Green CF, Mottram DR, Pirmohamed M. Adverse drug reactions in hospitals: a narrative review. Curr Drug Saf. 2007;2(1):79–87. doi:10.2174/157488607779315507. 18690953

[11] Jarzyna D, Jungquist CR, Pasero C, Willens JS, Nisbet A, Oakes L, Dempsey SJ, Santangelo D, Polomano RC. American society for pain management nursing guidelines on monitoring for opioid-induced sedation and respiratory depression. Pain Manag Nurs. 2011;12(3):118–145 e110. doi:10.1016/j.pmn.2011.06.008. 21893302

[12] Gupta K, Prasad A, Nagappa M, Wong J, Abrahamyan L, Chung FF. Risk factors for opioid-induced respiratory depression and failure to rescue: a review. Curr Opin Anesthesio. 2018;31(1):110–19. doi:10.1097/ACO.0000000000000541. 29120929

[13] Pawasauskas J, Stevens B, Youssef R, Kelley M. Predictors of naloxone use for respiratory depression and oversedation in hospitalized adults. Am J Health-Syst Ph. 2014;71(9):746–50. doi:10.2146/ajhp130568. 24733138

[14] von Elm E, Altman DG, Egger M, Pocock SJ, Gøtzsche PC, Vandenbroucke JP. The strengthening the reporting of observational studies in epidemiology (STROBE) statement: guidelines for reporting observational studies. Int J Surg. 2014;12(12):1495–99. doi:10.1016/j.ijsu.2014.07.013. 25046131

[15] Faul F, Erdfelder E, Lang AG, Buchner A. G*Power 3: A flexible statistical power analysis program for the social, behavioral, and biomedical sciences. Behav Res Methods. 2007;39(2):175–91. doi:10.3758/BF03193146. 17695343

[16] Chen HN, Cohen P, Chen S. How big is a big odds ratio? Interpreting the magnitudes of odds ratios in epidemiological studies. Commun Stat-Simul C. 2010;39(4):860–64. doi:10.1080/03610911003650383.

[17] Pham PC, Khaing K, Sievers TM, Pham PM, Miller JM, Pham SV, Pham PA, Pham PT. 2017 update on pain management in patients with chronic kidney disease. Clin Kidney J. 2017;10(5):688–97. doi:10.1093/ckj/sfx080. 28979781 PMC5622905

[18] Dean M. Opioids in renal failure and dialysis patients. J Pain Symptom Manage. 2004;28(5):497–504. doi:10.1016/j.jpainsymman.2004.02.021. 15504625

[19] Tegeder I, Lotsch J, Geisslinger G. Pharmacokinetics of opioids in liver disease. Clin Pharmacokinet. 1999;37(1):17–40. doi:10.2165/00003088-199937010-00002. 10451781

[20] Pasero C, McCaffery M. Monitoring sedation - it’s the key to preventing opioid-induced respiratory depression. Am J Nurs. 2002;102(2):67–69. doi:10.1097/00000446-200202000-00026. 11953523

[21] Altamura AC, Moliterno D, Paletta S, Maffini M, Mauri MC, Bareggi S. Understanding the pharmacokinetics of anxiolytic drugs. Expert Opin Drug Met. 2013;9(4):423–40. doi:10.1517/17425255.2013.759209. 23330992

[22] Gudin JA, Mogali S, Jones JD, Comer SD. Risks, management, and monitoring of combination opioid, benzodiazepines, and/or alcohol use. Postgrad Med. 2013;125(4):115–30. doi:10.3810/pgm.2013.07.2684. PMC4057040 23933900

[23] Yung L, Lee KC, Hsu C, Furnish T, Atayee RS. Patterns of naloxone use in hospitalized patients. Postgrad Med. 2017;129(1):40–45. doi:10.1080/00325481.2017.1263139. 27858510

[24] Gordon DB, Pellino TA. Incidence and characteristics of naloxone use in postoperative pain management: a critical examination of naloxone use as a potential quality measure. Pain Manag Nurs. 2005;6(1):30–36. doi:10.1016/j.pmn.2004.12.003. 15917742

[25] Ramachandran SK, Haider N, Saran KA, Mathis M, Kim J, Morris M, O’Reilly M. Life-threatening critical respiratory events: a retrospective study of postoperative patients found unresponsive during analgesic therapy. J Clin Anesth. 2011;23(3):207–13. doi:10.1016/j.jclinane.2010.09.003. 21570616

[26] Taylor S, Kirton OC, Staff I, Kozol RA. Postoperative day one: a high risk period for respiratory events. Am J Surg. 2005;190(5):752–56. doi:10.1016/j.amjsurg.2005.07.015. 16226953

[27] Nadpara PA, Joyce AR, Murrelle EL, Carroll NW, Carroll NV, Barnard M, Zedler BK. Risk factors for serious prescription opioid-induced respiratory depression or overdose: comparison of commercially insured and veterans health affairs populations. Pain Med. 2018;19(1):79–96. doi:10.1093/pm/pnx038. 28419384 PMC5939871

[28] Zedler B, Xie L, Wang L, Joyce A, Vick C, Kariburyo F, Rajan P, Baser O, Murrelle L. Risk factors for serious prescription opioid-related toxicity or overdose among Veterans Health Administration patients. Pain Med. 2014;15(11):1911–29. doi:10.1111/pme.12480. 24931395

[29] Overdyk F, Dahan A, Roozekrans M, van der Schrier R, Aarts L, Niesters M. Opioid-induced respiratory depression in the acute care setting: a compendium of case reports. Pain Manag. 2014;4(4):317–25. doi:10.2217/pmt.14.19. 25300390

[30] Jungquist CR, Card E, Charchaflieh J, Gali B, Yilmaz M. Preventing opioid-induced respiratory depression in the hospitalized patient with obstructive sleep apnea. J Perianesth Nurs. 2018;33(5):601–07. doi:10.1016/j.jopan.2016.09.013. 30236566

[31] Fine PG, Mahajan G, McPherson ML. Long-acting opioids and short-acting opioids: appropriate use in chronic pain management. Pain Med. 2009;10:S79–S88. doi:10.1111/j.1526-4637.2009.00666.x. 19691687

[32] Rzasa Lynn R, Galinkin JL. Naloxone dosage for opioid reversal: current evidence and clinical implications. Ther Adv Drug Saf. 2018;9(1):63–88. doi:10.1177/2042098617744161. 29318006 PMC5753997

[33] Manglik A, Lin H, Aryal DK, McCorvy JD, Dengler D, Corder G, Levit A, Kling RC, Bernat V, Hübner H, et al. Structure-based discovery of opioid analgesics with reduced side effects. Nature. 2016;537(7619):185–90. doi:10.1038/nature19112. 27533032 PMC5161585

[34] Jungquist CR, Quinlan-Colwell A, Vallerand A, Carlisle HL, Cooney M, Dempsey SJ, Dunwoody D, Maly A, meloche K, Meyers A, et al. American society for pain management nursing guidelines on monitoring for opioid-induced advancing sedation and respiratory depression: revisions. Pain Manag Nurs. 2019;21(1):7–25. doi:10.1016/j.pmn.2019.06.007. 31377031

