# Supplementary material for: Risk factors for severe opioid-induced respiratory depression in hospitalized adults: A case–control study
Source: Can J Pain. 2020 May 21;4(1):103–10. doi: 10.1080/24740527.2020.1714431 (PMC7951145; doi:10.1080/24740527.2020.1714431)
Supplement: Supplemental Material [file UCJP_A_1714431_SM6000.docx]

**Supplemental File 1. Definition and measurement of predictor variables**

| Variables | Definition | Measurement |
| --- | --- | --- |
| Age | Age documented during the hospitalization | Years |
| Body Mass Index | Body mass index calculated using the weight in kg and height in m. | kg/m2 |
| Substance use disorder | Documentation of drug addiction during the respective admission | Presence  Absence |
| Alcohol use disorder | Patient identified as alcoholic in the medical file. | Presence  Absence |
| Sleep apnea | Patient known for sleep apnea. | Presence  Absence |
| Retrognathia | A malocclusion referring to an abnormal posterior positioning of the maxilla or mandible | Presence  Absence |
| Neck circumference | The measurement of the neck circumference in cm. | cm |
| Smoking | Documentation of smoking status during the hospitalization. | Presence (past or current smoker)  Absence (never smoked) |
| Respiratory disease | Asthma; chronic obstructive pulmonary disease; metastases; pleural effusion; pulmonary hypertension; atelectasis; pneumonia; pulmonary embolism; interstitial lung disease | Presence  Absence |
| Cardiac disease | Congestive heart failure; coronary artery disease; arrhythmia; aortic stenosis; myocardial infarction; pericarditis | Presence  Absence |
| Renal failure | Documentation of renal failure. | Presence  Absence |
| Liver failure | Documentation of liver failure. | Presence  Absence |
| Neurologic disorder | Multiple sclerosis; Guillan-Barre Syndrome; Parkinson’s Disease; head injury; subdural hemorrhage; cerebrovascular accident; transient ischemic attack; encephalopathy; brain tumour; epilepsy; metastases; dementia; confusion; cognitive impairment;  fluctuating level of consciousness; altered mental status; spinal compression; spina bifida | Presence  Absence |
| Psychiatric disorder | Schizophrenia; bipolar disorder; borderline personality disorder; conversion disorder; post-traumatic stress disorder; anxiety disorder; depression; paranoia | Presence  Absence |
| Mobility restrictions | Braden Scale (Mobility): 1=Completely immobile (Does not make even slight changes in body or extremity position without assistance), 2= Very limited (Makes occasional slight changes in body or extremity position, Unable to make frequent or significant changes independently), 3= Slightly limited (Makes frequent though slight changes in body or extremity position independently), 4=No limitations (Makes major and frequent changes in position without assistance. | Presence (very limited or completely immobile)  Absence (slightly limited or no limitations) |
| American Society of Anesthesiologists status classification | American Society of Anesthesiologists (ASA) physical status score documented on the operative record. | Score |
| Opioid naive | Patients who require a high dose of opioid in short period of time (e.g. 10 mg IV morphine or equivalent in post-anesthesia care unit | Presence  Absence |
| First 24h opioid | Cases who experienced opioid-induced respiratory depression during their first 24 hours of opioid administration. Data extraction for controls covered their first 24 hours of opioid administration. | Presence  Absence |
| Central nervous system (CNS) depressants | Concomitant administration of central nervous system depressants such as: Sedative; anticholinergic; antidepressant; antihistamine; benzodiazepine; muscle relaxant; anticonvulsant; antiemetic; antipsychotic; hypnotics; ketamine | Presence  Absence |
| Large single bolus | Large single-bolus techniques for opioid administration (e.g., single-injection neuraxial morphine) | Presence  Absence |
| Past naloxone | Previous naloxone administration regardless of timing. | Presence  Absence |
| Short episode of severe pain | Patients experienced pain that stopped suddenly (e.g., renal colic) | Presence  Absence |
| Length of surgery | Duration of surgical procedure. | Hours and minutes |
| Large incisions | Thoracic or abdominal incisions that may interfere with adequate ventilation. | Presence  Absence |
| Long + short acting | Patients received at least one short acting opioid in addition to a topical or long-acting opioid. | Presence  Absence |
| Change in opioid molecule | The opioid molecule was changed. | Presence  Absence |
| Increased opioid dose | The opioid dose was increased by at least 50% compared to the preceding dose. | Presence  Absence |
| Per os to parenteral | The route of administration changed from per os to parenteral (i.e., subcutaneous (SC), intramuscular (IM), intravenous (IV)) | Presence  Absence |
| SC, IM to IV, epidural | The route of administration changed from SC, IM to IV or epidural. | Presence  Absence |
| Total opioid over 24 h | The total dose of opioid received during the 24-h data extraction time frame measured in mg of Morphine per os. | mg Morphine per os |
