# Supplementary material for: Risk factors for severe opioid-induced respiratory depression in hospitalized adults: A case–control study
Source: Can J Pain. 2020 May 21;4(1):103–10. doi: 10.1080/24740527.2020.1714431 (PMC7951145; doi:10.1080/24740527.2020.1714431)
Supplement: Supplemental Material [file UCJP_A_1714431_SM5999.docx]

**Supplemental File 2. Risk factors for respiratory depression in hospitalized adults.**

| Risk factor | N included in analysis | N with risk factor present  (n, %) | Prevalence | | Level of significance  (p) | Odds Ratio | 95 % CI | Nagelkerke R2 |
| --- | --- | --- | --- | --- | --- | --- | --- | --- |
|  |  |  | Controls  (n, %) | Cases  (n, %) |  |  |  |  |
| Age | 266 | - | - | - | 0.001* | 1.025 | 1.01-1.04 | 5.9% |
| Body mass index (kg/m^2^) | 68 | - | - | - | 0.522 | 0.974 | 0.89-1.06 | 0.8% |
| Substance use disorder | 266 | 9 (3.4) | 3 (2.3) | 6 (4.5) | 0.318 | 2.047 | 0.5-8.36 | 0.5% |
| Alcohol use disorder | 266 | 9 (3.4) | 4 (3.0) | 5 (3.8) | 0.735 | 1.260 | 0.33-4.79 | 0.1% |
| Sleep apnea | 266 | 11 (4.1) | 4 (3.0) | 7 (5.3) | 0.362 | 1.792 | 0.51-6.27 | 0.4% |
| Smoking | 266 | 64 (24.1) | 35 (26.3) | 29 (21.8) | 0.390 | 0.781 | 0.44-1.37 | 0.4% |
| Respiratory disease | 266 | 90 (33.8) | 37 (27.8) | 53 (39.8) | 0.039* | 1.719 | 1.03-2.87 | 2.1% |
| Cardiac disease | 266 | 100 (37.6) | 40 (30.1) | 60 (45.1) | 0.012* | 1.911 | 1.15-3.16 | 3.2% |
| Renal failure | 266 | 46 (17.3) | 13 (9.8) | 33 (24.8) | 0.002* | 3.046 | 1.52-6.10 | 5.3% |
| Liver failure | 266 | 21 (7.9) | 6 (4.5) | 15 (11.3) | 0.048* | 2.691 | 1.01-7.17 | 2.1% |
| Neurologic disorder | 266 | 72 (27.1) | 30 (22.6) | 42 (31.6) | 0.099 | 1.585 | 0.92-2.74 | 1.4% |
| Psychiatric disorder | 266 | 31 (11.7) | 19 (14.3) | 12 (9.0) | 0.184 | 0.595 | 0.28-1.28 | 0.9% |
| Mobility restrictions | 240 | 95 (39.6) | 39 (33.6) | 56 (45.2) | 0.068 | 1.626 | 0.96-2.74 | 1.8% |
| American Society of Anesthesiologists score | 74 | - | - | - | 0.096 | 0.702 | 0.46-1.07 | 5.2% |
| Opioid naive | 266 | 28 (10.5) | 13 (9.8) | 15 (11.3) | 0.690 | 1.173 | 0.54-2.57 | 0.1% |
| First 24h opioid | 266 | 91 (34.2) | 37 (27.8) | 54 (40.6) | 0.029* | 1.774 | 1.06-2.96 | 2.4% |
| Central nervous system depressants | 266 | 176 (66.2) | 79 (59.4) | 97 (72.9) | 0.020* | 1.842 | 1.10-3.09 | 2.7% |
| Sedative | 266 | 10 (3.8) | 4 (3.0) | 6 (4.5) | 0.522 | 1.524 | 0.42-5.53 | 0.2% |
| Anticholinergic | 266 | 2 (0.8) | 1 (0.8) | 1 (0.8) | 1.00 | 1.00 | 0.06-16.2 | - |
| Antidepressant | 266 | 14 (5.3) | 4 (3.0) | 10 (7.5) | 0.111 | 2.622 | 0.80-8.58 | 1.4% |
| Antihistamine | 266 | 3 (1.1) | 1 (0.8) | 2 (1.5) | 0.569 | 2.015 | 0.18-22.5 | 0.2% |
| Benzodiazepine | 266 | 64 (24.1) | 24 (18.0) | 40 (30.1) | 0.023* | 1.953 | 1.10-3.48 | 2.6% |
| Muscle relaxant | 266 | 13 (4.9) | 6 (4.5) | 7 (5.3) | 0.776 | 1.176 | 0.38-3.59 | - |
| Anticonvulsant | 266 | 61 (22.9) | 25 (18.8) | 36 (27.1) | 0.110 | 1.603 | 0.9-2.86 | 1.3% |
| Antiemetic | 266 | 54 (20.3) | 27 (20.3) | 27 (20.3) | 1.00 | 1.00 | 0.55-1.82 | - |
| Antipsychotic | 266 | 24 (9.0) | 12 (9.0) | 12 (9.0) | 1.00 | 1.00 | 0.43-2.31 | - |
| Hypnotics | 266 | 13 (4.9) | 8 (6.0) | 5 (3.8) | 0.398 | 0.61 | 0.19-1.92 | 0.4% |
| Ketamine | 266 | 1 (0.4) | - | 1 (0.8) | 1.00 | - | - | 0.7% |
| Large single bolus | 266 | 1 (0.4) | - | 1 (0.8) | 1.00 | - | - | 0.7% |
| Past naloxone | 266 | 6 (2.3) | 1 (0.8) | 5 (3.8) | 0.137 | 5.156 | 0.59-44.7 | 1.5% |
| Short episodes of severe pain | 266 | 5 (1.9) | - | 5 (3.8) | 1.00 | - | - | 3.5% |
| Length of surgery | 80 | - | - | - | 0.148 | 1.00 | 1.00-1.00 | 3.8% |
| Large incision | 266 | 24 (9.0) | 16 (12.0) | 8 (6.0) | 0.093 | 0.468 | 0.19-1.13 | 1.5% |
| Long + short acting | 266 | 51 (19.2) | 31 (23.3) | 20 (15.0) | 0.089 | 0.582 | 0.31-1.09 | 1.5% |
| Change in opioid molecule | 266 | 57 (21.4) | 31 (23.3) | 26 (19.5) | 0.455 | 0.800 | 0.44-1.44 | 0.3% |
| Increased opioid dose | 266 | 26 (9.8) | 9 (6.8) | 17 (12.8) | 0.104 | 2.019 | 0.87-4.71 | 1.4% |
| Per os to parenteral | 266 | 28 (10.5) | 13 (9.8) | 15 (11.3) | 0.690 | 1.173 | 0.54-2.57 | 0.1% |
| Subcutaneous, intramuscular to intravenous, epidural | 266 | 2 (0.8) | - | 2 (1.5) | 1.00 | - | - | 1.4% |
| Total opioid over 24 h | 266 | - | - | - | 0.124 | 1.001 | 1.00-1.00 | 1.8% |

*p<0.05
